# Supplementary material for: Predicting visual function by interpreting a neuronal wiring diagram
Source: Nature. 2024 Oct 2;634(8032):113–23. doi: 10.1038/s41586-024-07953-5 (PMC11446822; doi:10.1038/s41586-024-07953-5)
Supplement: Supplementary file 6 — CRF and ERF predictions for individual TmY4 and TmY9 cells. Analogous to Supplementary Data 3, but for TmY target types. Shown are the top four monosynaptic pathways, the strongest pathway passing through each of the top ten intermediary types (ranking from Extended Data Fig. 7), and the trisynaptic pathway Tm1–TmY–Dm3–TmY (see the section entitled Prediction of spatial normalization). [file 41586_2024_7953_MOESM6_ESM.zip › DataS4/TmY9qΓèÑ/720575940639205208.pdf]

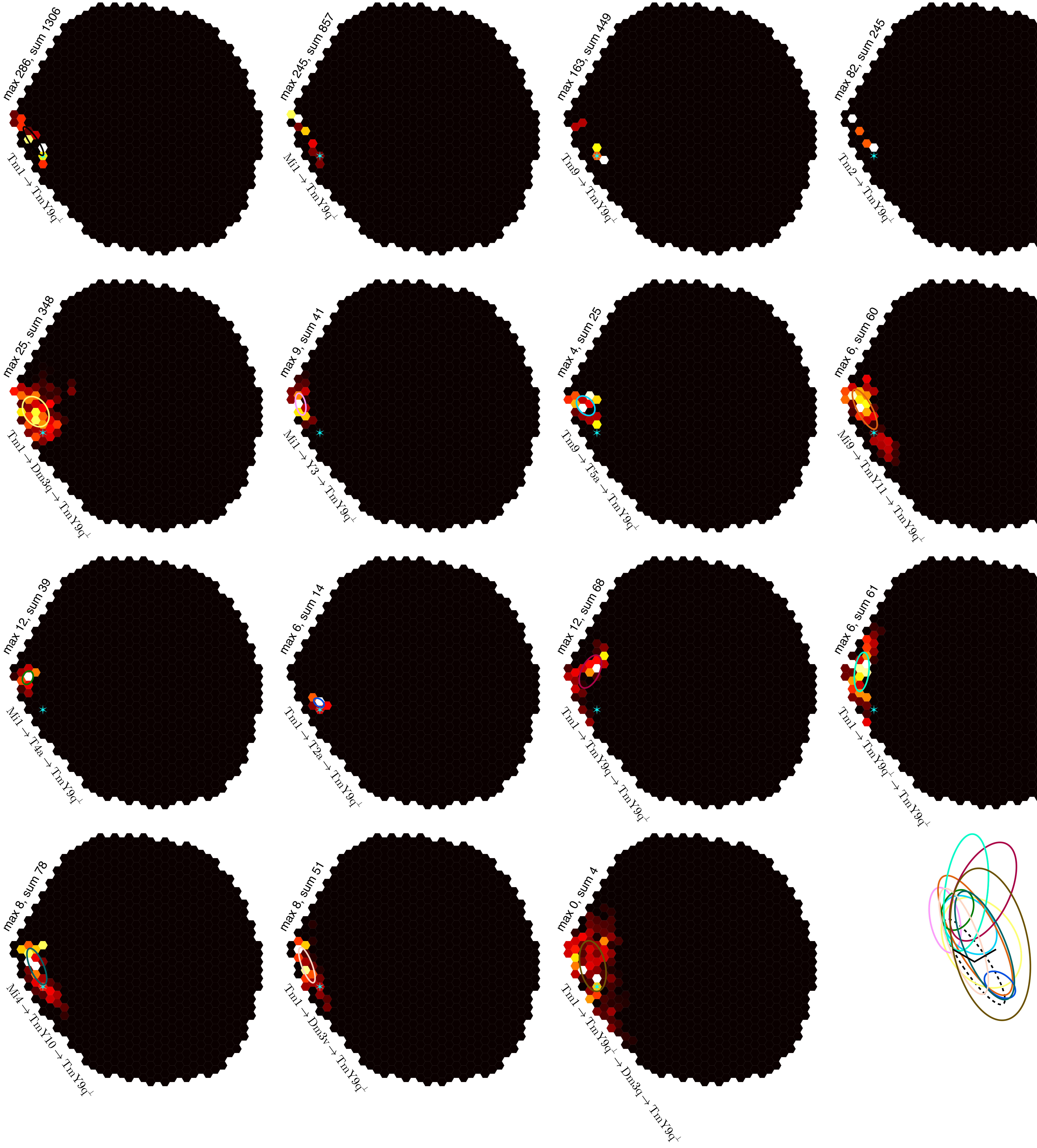
max 286, sum 1306
Tm1 → TmY9q⊥
max 245, sum 857
Mi1 → TmY9q⊥
max 163, sum 449
Tm9 → TmY9q⊥
max 82, sum 245
Tm2 → TmY9q⊥
max 25, sum 348
Tm1 → Dm3q → TmY9q⊥
max 9, sum 41
Mi1 → Y3 → TmY9q⊥
max 4, sum 25
Tm9 → T5a → TmY9q⊥
max 6, sum 60
Mi9 → TmY11 → TmY9q⊥
max 12, sum 39
Mi1 → T4a → TmY9q⊥
max 6, sum 14
Tm1 → T2a → TmY9q⊥
max 12, sum 68
Tm1 → TmY9q → TmY9q⊥
max 6, sum 61
Tm1 → TmY9q⊥ → TmY9q⊥
max 8, sum 78
Mi4 → TmY10 → TmY9q⊥
max 8, sum 51
Tm1 → Dm3v → TmY9q⊥
max 0, sum 4
Tm1 → TmY9q⊥ → Dm3q → TmY9q⊥
